# Geometry-complete latent diffusion model for 3D molecule generation

**DOI:** 10.1093/bioinformatics/btaf426

**Published:** 2025-07-30

**Authors:** Qunhao Zhang, Jun Xiao, Dongjiang Niu, Zhixin Zhang, Shanyang Ding, Zhen Li

**Affiliations:** College of Computer Science and Technology, Qingdao University, Shandong 266071, China; College of Computer Science and Technology, Qingdao University, Shandong 266071, China; College of Computer Science and Technology, Qingdao University, Shandong 266071, China; College of Computer Science and Technology, Qingdao University, Shandong 266071, China; College of Computer Science and Technology, Qingdao University, Shandong 266071, China; College of Computer Science and Technology, Qingdao University, Shandong 266071, China

## Abstract

**Motivation:**

Generative models, especially diffusion models, have recently made remarkable progress in fields such as graph generation and drug design. However, current diffusion-based 3D molecule generation models still struggle with adequately modeling the true data distribution.

**Results:**

We designed the geometry-complete latent diffusion model (GCLDM) to enhance the modeling capacity of diffusion models. A geometry-complete autoencoder for feature mapping between atom space and latent space is introduced. In addition, the latent space diffusion model can model continuous latent representations, which is helpful in learning to fit multi-modal feature distributions for the diffusion model. The comparative experimental results demonstrate that GCLDM could fit the true distribution of molecules well and outperform other state-of-art methods.

**Availability and implementation:**

Our codes and data are all provided at: [https://github.com/charlotte0104/GCLDM-for-3d-molucule-generation], and [https://zenodo.org/records/15773195].

## 1 Introduction

Traditional molecule design and discovery methods rely on experimental screening, theoretical calculations, and the expertise of chemists. However, these approaches often fall short when facing the vast molecule space and the complexity of biological systems. The development of deep learning methods speeds up the process of drug discovery. In addition, the number of compounds that have been synthesized is estimated to be about $10^{8}$, while the total number of theoretically feasible compounds ranges from $10^{23}$ to $10^{60}$ (Polishchuk *et al.* 2013). Therefore, there is an urgent need to explore the large chemical space to solve problems in chemistry, pharmacology (Bilodeau *et al.* 2022).

Deep generative model offers a viable solution to the above challenges (Pereira *et al.* 2016). By learning the distribution of various compound molecules through deep generative modeling, stable and robust molecules can be sampled from the generative model. The method for molecule generation could be categorized into five types, i.e. autoregressive models, variational autoencoders, flow-based models, generative adversarial networks, and diffusion models. Autoregressive models treat the generative process as a series of steps, using the output of the previous step to predict the output of the next step. For example, GraphRNN (You *et al.* 2018) represents graphs with different node orders as sequences and utilizes two recurrent neural networks to learn the distribution of a large number of graph representations varying with node order, where the graph-level RNN adds a node to the graph at each step, and the edge-level RNN generates connectivity relations between the new nodes and the known nodes. GraphVAE (Simonovsky and Komodakis 2018) is a graph generation model based on the variational autoencoder, which consists of an encoder that maps the input data to a low-dimensional latent space and a decoder that maps the latent representation back to the original data space. GraphVAE solves the edge generation problem by training the decoder to output a probabilistic fully connected graph of the reconstructed graph. GraphNVP (Madhawa *et al.* 2019) is designed based on normalized flow, which models complex probability distributions by an encoder, while the decoder is designed as an inverse function of the encoder. GraphNVP proposes a two-step generative scheme, which first generates a graph structure and then generates attributes of nodes based on the structure, thus solving the problem of difficulty in generating edge information caused by the arbitrary connectivity of graphs. molGAN (Cao and Kipf 2022) is designed for the generation of small molecule graphs based on generative adversarial networks, which consists of a generator and a discriminator. The generator is used to generate samples that deceive the discriminator, while the discriminator is used to evaluate the authenticity of the data generated by the generator. In addition, molGAN guides the model to generate more robust molecules by introducing a reinforcement learning loss in the discriminator’s objective function.

In recent years, the diffusion model has been applied as a new type of generative model for generative tasks (Anand and Achim 2022, Lin *et al.* 2025). The diffusion model defines a process of progressively perturbing the data with noise and reversing the above process by a neural network that learns to denoise, which can generate high-quality, realistic samples. The most representative diffusion model applied to the molecule generation is E(3) Equivariant Diffusion Model (EDM) (Hoogeboom *et al.* 2022). EDM is designed based on denoising diffusion probabilistic model, which represents molecules as atom coordinates and atom features. It treats the molecule graph as a fully connected graph in the learning phase, thus ignoring the effect brought by the edge information, while in the sampling stage, EDM generates only the position information of the nodes and atom features. The chemical bonds are then predicted based on the atom position information of the generated samples, thus obtaining a complete and robust representation of the molecule. GeoLDM (Xu *et al.* 2023) is an improvement of EDM by performing the mapping of features in atom space and latent space through an EGNN (Satorras *et al.* 2021) autoencoder (Kingma 2013) while placing an EDM in the latent space (Rombach *et al.* 2022). MUDiff (Hua *et al.* 2024), also a DDPM-based molecule generative model, designed a two-channel graph transformer (Yun *et al.* 2019) including a 2D invariant channel and a 3D equivariant channel to process 2D and 3D information, respectively. It is able to denoise the complete molecule representation simultaneously, and directly generate the complete molecule representation including atom features, 2D discrete molecule structures, and 3D continuous molecule coordinates.

However, there are still some problems with the current methods. Firstly, the necessary information contained in a molecule includes atom coordinates, atom types, atom charge, and physicochemical properties, which means that a molecule can be considered as consisting of multiple features, In atom space, the diffusion model’s ability is limited when faced with multi-modal features because of the uniform Gaussian diffusion framework (Wu *et al.* 2022, Xu *et al.* 2023).

Second, molecule 3D structural information is to the benefit of molecule generation, Therefore, how to effectively utilize the 3D position information of the graph is also a problem to solve. However, conventional graph convolution cannot be used in the diffusion model on 3D graph directly. The equivariant graph neural networks provide a solution to the above problems, which treats the input as a fully connected graph and introduces the Euclidean distances between atoms as an input to the message passing paradigm of graph neural networks. E(3) equivariant graph transformer (Thölke and De Fabritiis 2022) introduces the atom Euclidean distances to the attention. ClofNet (Du *et al.* 2022) incorporates equivariant local complete frames into graph neural networks to efficiently approximate the geometric quantities. The SE(3) equivariant geometry-complete perceptual network also introduces geometry-complete frames in the message passing process of graph neural networks, and introduces geometry-complete perception modules to enable the model to learn directly from 3D molecular graphs (Morehead and Cheng 2024), which has been proven to have better results in dealing with chiral molecules. Although GeoLDM (Xu *et al.* 2023) uses EGNN-based E(3) equivariant model as autoencoder to strengthen the diffusion model’s ability to learn the distribution of training set, its insensitivity to mirror transformation in Euclidean space and lack of learning ability for 3D molecules still affect the performance of the molecule generation.

In order to solve the above problems, a geometry-complete latent diffusion model (GCLDM) is proposed in this paper. A SE(3) equivariant geometry-complete graph autoencoder with geometry-complete message passing is introduced in GCLDM, where atom features are first mapped from discrete space to continuous latent space by geometry-complete perceptron convolution. The diffusion model is used to learn the distribution of continuous features in the latent space. In addition, the geometry-complete autoencoder can reduce the information loss in the encoding process with the help of its sensitivity to mirror transformation and geometry-complete message passing mechanism, which is beneficial for latent diffusion to model the distribution of latent representations more accurately. The GCLDM is evaluated on several benchmarks, for unconditional generation and conditional generation to demonstrate the validity of the model.

## 2 Materials and methods

### 2.1 Overview

**Figure 1.**
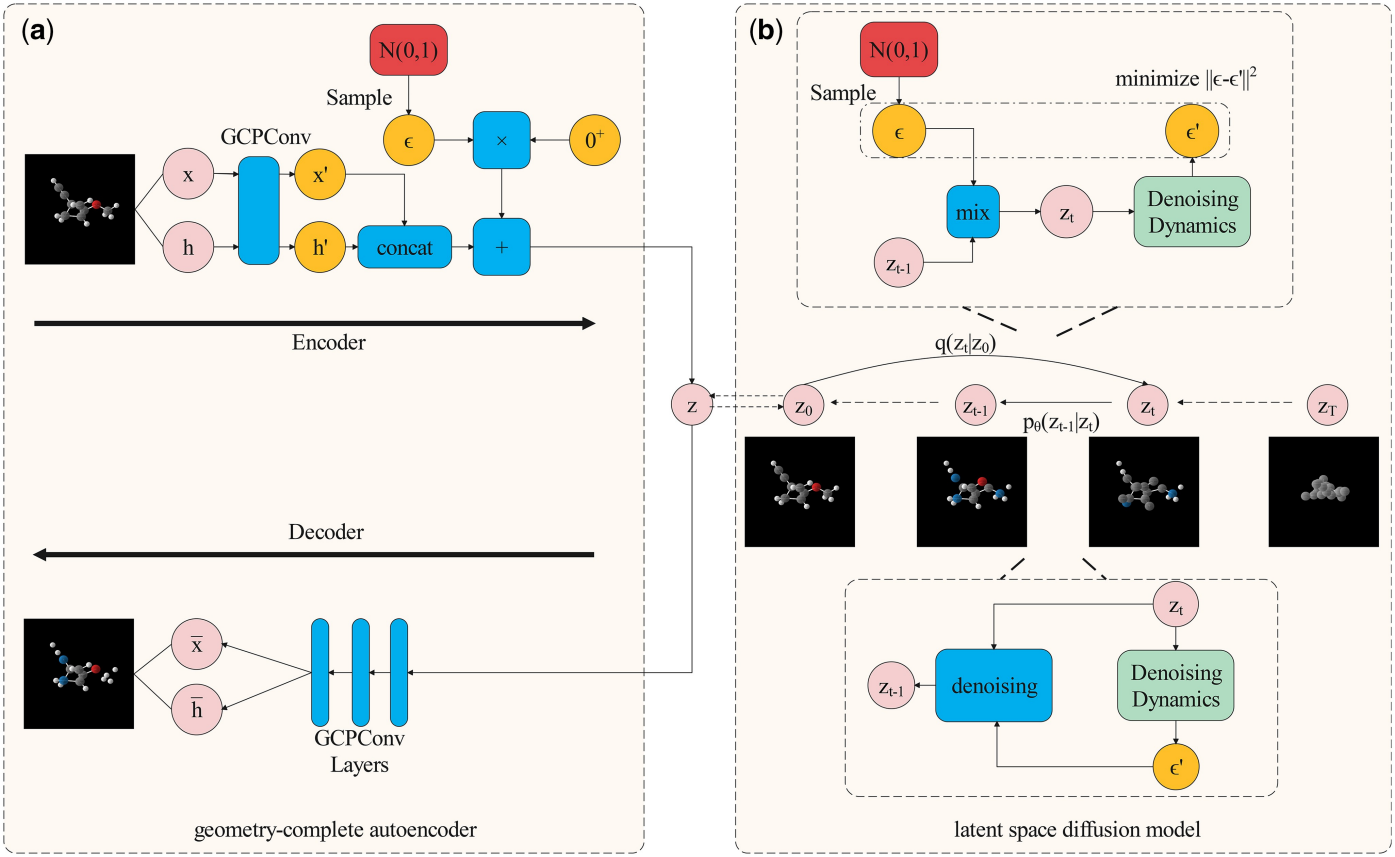
The framework of GCLDM. (a) Geometry-complete autoencoder and (b) latent space diffusion model.

The overall framework of GCLDM is shown in Fig. 1, which consists of a geometry-complete autoencoder and a latent space diffusion model. The encoder maps the multi-modal atom coordinates and features to a continuous latent space through a geometry-complete perceptron convolution layer, and the decoding process maps the latent representation of the 3D molecule back to the atom space to form a robust molecule. The latent space diffusion is then implemented based on a denoising diffusion probabilistic model, with geometry-complete perceptron convolution used to parameterize the denoising dynamics of the diffusion model.

### 2.2 Geometry-complete autoencoder

#### 2.2.1 Geometry-complete perceptron convolution

Given a graph G=(V,E), *V* and *E* denote the set of nodes and the set of edges of the graph *G*, respectively, N=|V| denotes the number of nodes of the graph, $x=(x_{1},x_{2},\ldots ,x_{N})\in R^{N\times 3}$ denote the coordinates of the nodes in 3D space. The nodes are characterized by scalar features $h\in R^{N\times nf}$ and vector features $\chi \in R^{N\times m}$, and all edges can be represented as scalar features $e\in R^{\left|E\right|\times ef}$ and vector features $\xi \in^{E\times k}$. Both vector features are calculated from the coordinates of the atoms. *nf* and *ef* denote the length of the scalar feature of each node and edge, respectively. For each node *i*, $h_{i}\in h$, $\chi_{i}\in \chi$, $e_{ij}\in e$ and $\xi_{ij}\in \xi$, geometry-complete message passing can be defined as (Morehead and Cheng 2024):


(1) $${\text{msg}}_{ij}=\Omega_{\omega }^{l}\left(n_{i}^{l-1},n_{j}^{l-1},e_{ij}^{l-1},\xi_{ij}^{l-1},F_{ij}\right)$$

(2) $${\text{msg}}_{i}=A_{\forall \;j\in \text{NBR}\left(i\right)}\left({\text{msg}}_{ij}\right)$$

(3) $$n_{i}^{l}=\phi^{l}\left(n_{i}^{l-1},{\text{msg}}_{i}\right)$$

(4) $$F_{ij}=\left(a_{ij},b_{ij},c_{ij}\right),$$

where $n_{i}^{l}=(h_{i}^{l},\chi_{i}^{l})$, $a_{ij}=\frac{x_{i}-x_{j}}{∥x_{i}-x_{j}∥}$, $b_{ij}=\frac{x_{i}\times x_{j}}{∥x_{i}\times x_{j}∥}$, $c_{ij}=a_{ij}\times b_{ij}$, $\phi^{l}$ is a trainable network and *l* denotes the depth of the current layer in the network, *A* is the aggregation function, and $\Omega_{\omega }$ denotes the geometry-complete message passing function, which computes the messages of the graph neural network from the vector and scalar features of nodes and the geometry-complete local frames $F_{ij}$. The coordinate of each atom could be updated through a simple SE(3) equivariant approach as follows:


(5) $$\left(h_{p_{i}}^{l},\chi_{p_{i}}^{l})={\text{GCP}}_{p}^{l}(n_{i}^{l},F_{ij}\right)$$

(6) $$x_{i}^{l}=x_{i}^{l-1}+\chi_{p_{i}}^{l},$$

where ${\text{GCP}}_{p}^{l}$ is called the geometry-complete perceptual module, which provides invariant updates $h_{p_{i}}^{l}$ for atom scalar features and vector feature updates $\chi_{p_{i}}^{l}$ that are equivariant to rotational transformation, so geometry-complete perceptual convolution (GCPConv) can use $\chi_{p_{i}}^{l}$ to update the atom coordinates. The geometry-complete message passing and geometry-complete perceptron module constitute the GCPConv.

In the above process, the χ, ξ, $F_{ij}^{t}$ are all calculated from the coordinates of the atoms in the current layer, and e is considered the fully connected adjacency matrix, which will not be changed across all layers, so the geometry-complete perceptual convolution can be simplified as the following equation:


(7) $$\left(x^{l},h^{l}\right)=\text{GCPConv}\left(x^{l-1},h^{l-1}\right)$$

Therefore, the representation of the graph in the following could also be described as G=(x,h). Moreover, it is demonstrated that the geometry-complete perceptron convolution meets the requirements of SE(3) equivariance (Serre *et al.* 1977):


(8) $$\left(Rx^{l}+\tau ,h^{l}\right)=\text{GCPConv}\left(Rx^{l-1}+\tau ,h^{l-1}\right),\;\forall R\;\text{and}\;\tau ,$$

where *R* denotes the rotational transformation in Euclidean space and τ denotes the translational transformation.

#### 2.2.2 Implementation of geometry-complete autoencoder

The significance of the autoencoder is that the cross-modal features can be compressed into the low-dimensional latent space, so that the discrete atom space features can become continuous in the latent space, which enables the diffusion model to learn the distribution of the training set more accurately. The autoencoder is divided into two modules, the encoder *E* and the decoder *D* (Kingma 2013), where the encoder encodes the graph *G* as a latent space representation z=E(x,h), and the decoder decodes the latent representation back to the atom space, which can be described as $\bar{x},\bar{h}=D\left(z\right)$. The whole autoencoder can be trained by optimizing the reconstruction loss.

A model that satisfies the SE(3) equivariant constraint is able to detect the effects of molecular chirality more accurately than the E(3) equivariant approach, since the model satisfying the E(3) equivariant constraint is insensitive to mirror transformations in space. In addition, it is demonstrated that geometrically complete message passing has a stronger learning ability for three-dimensional molecules. Therefore, a new SE(3) autoencoder is designed in GCLDM based on the geometry-complete perceptron network, as shown in Fig. 1a. The encoding process $z=\left(z_{x},z_{h}\right)=E\left(x,h\right)$ can be summarized as:


(9) $$\;\left(z_{x},z_{h}\right)=\text{GCPConv}\left(x,h\right)+\sigma \times \delta ,$$

where $z_{x}$, $z_{h}$ are the representations of the coordinates and scalar features in latent space, respectively, σ is the noise sampled from the standard normal distribution, and δ is a positive value close to zero. The decoding operation can be described as:


(10) $$\left(\bar{x},\bar{h}\right)=\text{GCPConv}\left(\text{GCPConv}\left(\text{GCPConv}\left(z_{x},z_{h}\right)\right)\right),$$

where we perform three geometry-complete perceptron convolutions during the decoding process with the aim of enhancing the decoder’s restoration capability just as shown in Fig. 1a, and $\bar{x}$, $\bar{h}$ denotes reconstructed coordinates and scalar features. The L2 norm is used as the loss of the autoencoder $L_{\text{AE}}$ as follows:


(11) $$L_{\text{AE}}=||(x,h)-\left(\bar{x},\bar{h}\right)|{|}^{2}$$

### 2.3 Latent space diffusion model

After being processed by the encoder, the diffusion model (Ho *et al.* 2020) could be implemented on the latent representations through a forward diffusion process. For each sampling step, the diffusion model samples noise from a standard normal distribution, and relies on the noise reduction capabilities of denoising dynamics to progressively denoise the samples to obtain the latent representation of the molecule.

The diffusion model can be described using two Markov chains. The forward Markov chain describes the process of progressively adding noise to the latent representation $z_{0}$ to obtain $z_{T}$. This can be expressed with the conditional probability $q\left(z_{1:T}|z_{0}\right)=\prod_{t=1}^{T}q\left(z_{t}|z_{t-1}\right)$. The reverse Markov chain, which corresponds to the denoising process, can be defined as $p_{\theta }\left(z_{0:T}\right)=p\left(z_{T}\right)\prod_{t=1}^{T}p_{\theta }\left(z_{t-1}|z_{t}\right)$. The diffusion model adds noise to the sample step by step through forward diffusion:


(12) $$q\left(z_{t}|z_{t-1}\right)=N\left(z_{t};\sqrt{1-\beta_{t}}z_{t-1},\beta_{t}I\right)$$

(13) $$q\left(z_{t}|z_{0}\right)=N\left(z_{t};\sqrt{{\bar{\alpha }}_{t}}z_{0},\left(1-{\bar{\alpha }}_{t}\right)I\right)$$

where $\beta_{1:t}$ controls the noise added to the data at each time step, and the sample $z_{T}$ approximately follows a standard normal distribution, i.e., $q\left(z_{T}\right)=N\left(0,I\right)$. $\alpha_{t}$ is a preset hyperparameter with $\alpha_{t}=1-\beta_{t}$, $\bar{\alpha_{t}}=\prod_{i=1}^{T}\alpha_{i}$. All parameters of the forward process are predefined and are not trainable. The generation process of a diffusion model is a learnable reverse denoising process, aiming to denoise the noisy variables $z_{T:1}$ to approximate the clean sample $a_{0}$ through the following equation:


(14) $$p_{\theta }\left(z_{t-1}|z_{t}\right)=N\left(z_{t-1};\mu_{\theta }\left(z_{t},t\right),\rho_{t}^{2}I\right),$$

where $\mu_{\theta }$ is the mean of the distribution which is the output of the trainable neural network. The variance $\rho_{t}^{2}$ is typically assumed to be the same as that of the forward diffusion process.

According to Bayes’ theorem:


(15) $$\begin{array}{c} q\left(z_{t-1}|z_{t},z_{0}\right)=\frac{q\left(z_{t}|z_{t-1}\right)q\left(z_{t-1}|z_{0}\right)}{q\left(z_{t}|z_{0}\right)}\\ =N(z_{t-1};\frac{1}{\sqrt{\alpha_{t}}}z_{t}-\frac{1-\alpha_{t}}{\sqrt{1-{\bar{\alpha }}_{t}}\sqrt{\alpha_{t}}}\epsilon ,\\ \;\frac{\left(1-\alpha_{t})(1-{\bar{\alpha }}_{t-1}\right)}{1-{\bar{\alpha }}_{t}}I) \end{array}$$

where ϵ∼N(0,I) is the noise randomly sampled from a standard normal distribution. The diffusion model typically uses the neural network $\mu_{\theta }$ to directly predict the random noise ϵ. As shown in the top section of Fig. 1b, its loss $L_{LD}$ can be defined as (Song and Ermon 2019, Ho *et al.* 2020):


(16) $$L_{\text{LD}}=||\epsilon -\mu_{\theta }(z_{t},t)|{|}^{2}$$

### 2.4 Geometry-complete latent diffusion

Compared to conventional image or text generation models, generating 3D geometric structures faces a challenge in that the latent representations *z* include not only invariant scalar features $z_{h}$ but also equivariant coordinate features $z_{x}$. This requires the distribution $p_{\theta }$ of diffusion models to satisfy the condition of critical invariance (Xu *et al.* 2023):


(17) $$p_{\theta }(z_{x},z_{h})=p_{\theta }(Rz_{x},z_{h}),\forall R$$

The related work in GeoDiff (Xu *et al.* 2022) demonstrates that the invariance of the diffusion model distribution can be achieved when the initial distribution $p\left(z_{x,t},z_{h,t}\right)$ is invariant and $p_{\theta }\left(z_{x,t-1},z_{h,t-1}|z_{x,t},z_{h,t}\right)$ is equivariant. Additionally, inspired by EDM, in which the noise prediction network $\mu_{\theta }$ is designed using equivariant graph neural networks, and we parameterized the denoising dynamics with geometry-complete perceptron convolution. With the help of GCPConv, the diffusion model not only achieves SE(3) equivariance but also enables the denoiser to predict noise more accurately. This is because the geometry-complete perceptron convolution encodes local reference frames, embedding geometric information into each node’s local frame to support geometry-complete and chirality-aware message passing.

#### 2.4.1 Training of GCLDM

For latent space diffusion, the autoencoder and the diffusion model are jointly trained using the combination of losses $L_{\text{LD}}$ and $L_{\text{AE}}$ as shown in Equation (18), which is then optimized through gradient descent:


(18) $$L_{\text{GCLD}}=L_{\text{AE}}+L_{\text{LD}}$$

#### 2.4.2 Sampling of GCLDM

For the sampling process, as shown in the bottom part of Fig. 1b, noise is directly sampled from the standard normal distribution in the latent space. The diffusion model then denoises the random noise to latent representations, and the decoder restores them back to the atom space:


(19) $$z_{t-1}=\frac{1}{\sqrt{\alpha_{t}}}\left(z_{t}-\frac{1-\alpha_{t}}{\sqrt{1-{\bar{\alpha }}_{t}}}\mu_{\theta }\left(z_{t},t\right)\right)+\sigma_{t}\epsilon \;$$

(20) $$\langle \bar{x},\bar{h}\rangle =D(z_{0}),$$

where $\mu_{\theta }$ is the output of the noise prediction network, $\sigma_{t}=\sqrt{\frac{\left(1-\alpha_{t})(1-{\bar{\alpha }}_{t-1}\right)}{1-{\bar{\alpha }}_{t}}}$, and ϵ∼ N(0,I) is a random value sampled from a standard normal distribution.

The issue with this approach is that to sample noise from a standard normal distribution, we first need to determine how many atoms the molecule has. For the number of nodes *N*, we traverse the training set to obtain the distribution p(N). Then, we first sample N∼p(N), and subsequently generate latent representation in size *N*.

#### 2.4.3 Conditional generation

In drug discovery and materials design, the goal is often to find molecules that meet specific requirements, for example, generating molecules with a specific polarizability or heat capacity value. With conditional generation, researchers can directly generate molecules that satisfy these needs, without having to search aimlessly through the vast molecule space, which is more efficient than unconditional generation, and significantly shortens the research time, so we will introduce the method of conditional generation that GCLDM adopts.

Given a condition *c*, to generate x,h∼p(x,h|c), the unconditional GCLDM is extended for conditional generation. Before each training step, the latent representations$\left(z_{x},z_{h}\right)$ is concatenated with the condition *c*, and the noise is predicted using $\mu_{\theta }$ for $\left(z_{x},z_{h},c\right)$ (Van Den Oord *et al.* 2016) and compute the loss:


(21) $$L_{LD}=||\epsilon -\mu_{\theta }(z_{t},c,t)|{|}^{2}$$

Similarly, during conditional sampling, the same operation is performed. The process of sampling $z_{t-1}$ from $z_{t}$ can be summarized as follows:


(22) $$z_{t-1}=\frac{1}{\sqrt{\alpha_{t}}}\left(z_{t}-\frac{1-\alpha_{t}}{\sqrt{1-{\bar{\alpha }}_{t}}}\mu_{\theta }(z_{t},c,t)\right)+\sigma_{t}\epsilon$$

## 3 Results

### 3.1 Unconditional molecule generation

#### 3.1.1 Unconditional generation on QM9

The QM9 (Ramakrishnan *et al.* 2014) dataset contains descriptions of molecular properties and 3D atom coordinates for 130 000 small molecules. In our experiments, we train a GCLDM to generate molecules with 3D atom coordinates and atom types under an unconditional constraint. The QM9 dataset is divided into training, validation, and test partitions, consisting of 100k, 18k, and 13k molecules (Anderson *et al.* 2019), respectively.

For the fairness in the comparative experiment, we used the same evaluation metrics as EDM (Hoogeboom *et al.* 2022), which are atom stability and molecule stability. We sample 3D structures of molecules without edge information from the model, predict chemical bond information based on the distances between atoms, which is used to calculate whether the valence of each atom is correct. Atoms with correct valence are considered stable atoms. Similarly, if every atom in a molecule is stable, the molecule is considered as a stable molecule. Finally, we compute the atom stability and molecule stability for all samples. In addition, we also utilize RDKit (Landrum *et al.* 2013) to evaluate the validity and uniqueness of molecules and incorporate these metrics into our evaluation framework.

The proposed GCLDM is compared with several competitive baseline models. GraphVAE (Simonovsky and Komodakis 2018), a non-equivariant molecular generation model, was included in our baselines to demonstrate the advantages of equivariant models. Equivariant Normalizing Flows (Garcia Satorras *et al.* 2021) is a prior flow-based equivariant generative model for molecules. G-Schnet (Gebauer *et al.* 2019) is a molecule generation model based on autoregression. Equivariant diffusion model (EDM) (Hoogeboom *et al.* 2022), an implementation of DDPM with an EGNN serving as the denoising dynamics, represents a pioneering advancement in diffusion-based molecule generation. Geometrically Complete Diffusion Model (GCDM) (Morehead and Cheng 2023) is an implementation of DDPM that parameterizes denoising dynamics with geometry-complete perceptron convolution. Finally, GeoLDM is a latent space diffusion model based on EDM, which introduces an equivariant graph autoencoder based on EDM to enhance its performance.

Ten thousand molecules were generated for evaluation, and the process was repeated three times, and the mean and standard deviation of each metric were used as the results. The published results of the baselines were used for the comparison. As shown in Table 1, GCLDM demonstrates a clear advantage over previous methods across all unconditional generation metrics. Compared to GCDM, which also parameterizes denoising dynamics with geometry-complete perceptron convolution, GCLDM improves molecule stability by approximately 5%, with significant improvements in the other three metrics as well. Specifically, diffusion models in the latent space exhibit stronger modeling capabilities than those in atom space. Compared to GeoLDM, another latent diffusion model, GCLDM also shows significant improvements in molecule stability, validity, and uniqueness, with gains of 1%, 1.4%, and 0.8%, respectively. This highlights that geometry-complete message passing outperforms E(3)-equivariant message passing in terms of noise prediction and feature extraction of 3D graph information.

**Table 1.** Comparative results of atom stability, molecule stability, validity, and uniqueness.^a^

Method	Atoms stable (%)	Mol stable (%)	Valid (%)	Valid and unique (%)
GraphVAE	–	–	55.7	42.3
E-NF	85.0	4.9	40.2	39.4
G-Schnet	95.7	68.1	85.5	80.3
EDM	98.7±0.1	82.0±0.4	91.9±0.5	90.7±0.6
GCDM	98.7±0.0	85.7±0.4	94.8±0.2	93.3±0.0
GeoLDM	98.9±0.1	89.4±0.5	93.8±0.4	92.7±0.5
GCLDM	99.0±0.0	90.3±0.2	95.2±0.1	93.5±0.5

a A higher value indicates a better generation quality.

Our experimental results confirm that GCLDM demonstrates a remarkable improvement in modeling the distribution of small molecules compared to traditional flow-based models and autoregressive models. Additionally, the GCLDM also outperformed recent works such as EDM, GCDM, and GeoLDM. All of the above highlights the effectiveness of GCLDM in capturing the complex molecule structures in 3D space.

#### 3.1.2 Unconditional generation on drug-like molecules

To further demonstrate the modeling capability of GCLDM for larger drug-like molecules, a total of 11 951 molecules are selected from the ChEMBL (Zdrazil *et al.* 2024) database based on the criteria of containing no more than 12 heavy atoms (QM9 contains at most 9 heavy atoms) and violating no more than one of Lipinski’s “Rule of Five” (Lipinski *et al.* 2012). The corresponding 3D conformations of these molecules are generated using RDKit, and the dataset is divided into training, validation, and test sets in a 6:2:2 ratio. We selected the most competitive GCDM and GeoLDM as baselines and reproduced their experiments on this dataset. The atom stability, molecule stability, and validity were reported as evaluation metrics.

Two thousand molecules were generated from each method for evaluation. The process was repeated three times, and the mean and standard deviation of each metric were used as the results. As shown in Table 2, GCLDM demonstrated higher atom and molecule stability on this dataset. Although its validity was slightly lower, the comprehensive evaluation indicated that GCLDM still achieved overall optimal results.

**Table 2.** Experimental results on drug-like molecular datasets.

Method	Atoms stable (%)	Mol stable (%)	Valid (%)
GeoLDM	92.03±0	39.68±0.3	75.53±0.3
GCDM	93.02±0.1	43.15±0.1	74.38±0.8
GCLDM	93.27±0.1	46.44±0.2	71.07±0.3

### 3.2 Conditional molecule generation

In this section, we aim to generate molecules targeting some desired properties.

The conditional molecule generation experiments were conducted on the QM9 dataset, where we trained and sampled molecules from the model conditionally with the polarizability α, dipole moment μ, heat capacity *Cv*, orbital energies $\epsilon_{\text{HOMO}}$, $\epsilon_{\text{LUMO}}$, and their gap Δϵ. To reasonably and fairly evaluate the model’s performance, we followed the evaluation process of EDM by splitting the QM9 training set into two halves: the second half was used to train the generative model, and the first half was used to train the EGNN classifier. After training, given a set of attribute values *c*, the generative model samples molecules that satisfy these conditions, and the classifier is used to predict the attribute values *pred* for this batch of samples. The mean absolute error (MAE) between *c* and *pred* is used as the evaluation metric for the experiment. A smaller error indicates better model performance.

For conditional generation of 3D molecules, three other diffusion models are used for comparison, including EDM, GCDM and GeoLDM. In addition, a lower-bound baseline is implemented by feeding QM9 samples into the EGNN classifier. The resulting predictions are used to calculate the MAE for performance evaluation. The results are shown in Table 3. A smaller gap compared to the lower-bound indicates a better property-conditioning performance.

**Table 3.** Mean absolute error for molecular property.^a^

Task	α **(**${\text{Bohr}}^{3}$**)**	μ **(D)**	$C_{v}$ $\left(\frac{\text{cal}}{\text{mol}}K\right)$	Δϵ **(meV)**	$\epsilon_{\text{HOMO}}$ **(meV)**	$\epsilon_{\text{LUMO}}$ **(meV)**
EDM	2.76	1.111	1.101	655	356	584
GCDM	1.97	0.844	0.689	602	344	479
GeoLDM	2.37	1.108	1.025	587	340	522
GCLDM	1.92	0.78	0.648	546	337	421
QM9	0.10	0.043	0.040	64	39	36

a A lower value indicates a better controllable generation result.

We conducted experiments treating each property as a condition, evaluating 10,000 samples for each method. Compared to GCDM and GeoLDM, GCLDM consistently achieves the smallest MAE on all properties. When compared to the previous results of GCDM and GeoLDM, GCLDM shows improvements of 2.5%, 7.5%, 5.9%, 6%, 0.8% and 12.1% for the molecular properties α, μ, *Cv*, Δϵ, $\epsilon_{\text{HOMO}}$ and $\epsilon_{\text{LUMO}}$, respectively, which demonstrates that GCLDM can model molecular properties more accurately.

### 3.3 Property distribution

**Figure 2.**
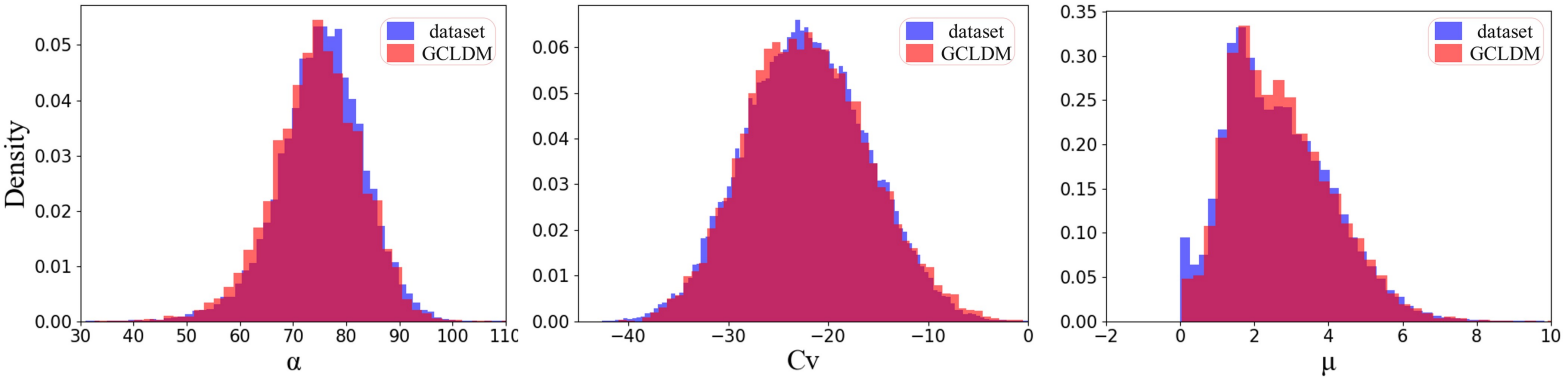
Property distribution of training set and GCLDM.

The molecule generation model is used to fit the data distribution of the training set to generate molecules. Therefore, the ability of GCLDM to model the molecular distribution can be reflected by the similarity of certain properties between its own distribution and the training set distribution, so we analyzed the distributions of properties such as polarizability, dipole moment, and heat capacity from molecules sampled from the unconditional GCLDM and compared them with the distributions of these properties in the QM9 training set. The probability density histograms are shown in Fig. 2. A higher similarity indicates a stronger modeling capability of the model.

As shown in Fig. 2, the distribution of GCLDM fits well with the dataset. For polarizability, dipole moment, and heat capacity, GCLDM captures the distributions of these properties very effectively, which validates the excellent modeling capability of GCLDM.

### 3.4 Visualization

In this section, we present visual demonstrations of the samples generated by GCLDM trained on QM9, including unconditional generation and conditional generation experiments. In addition, the process of GCLDM transforming random noise into robust molecules is also provided.

#### 3.4.1 Unconditional generation

**Figure 3.**
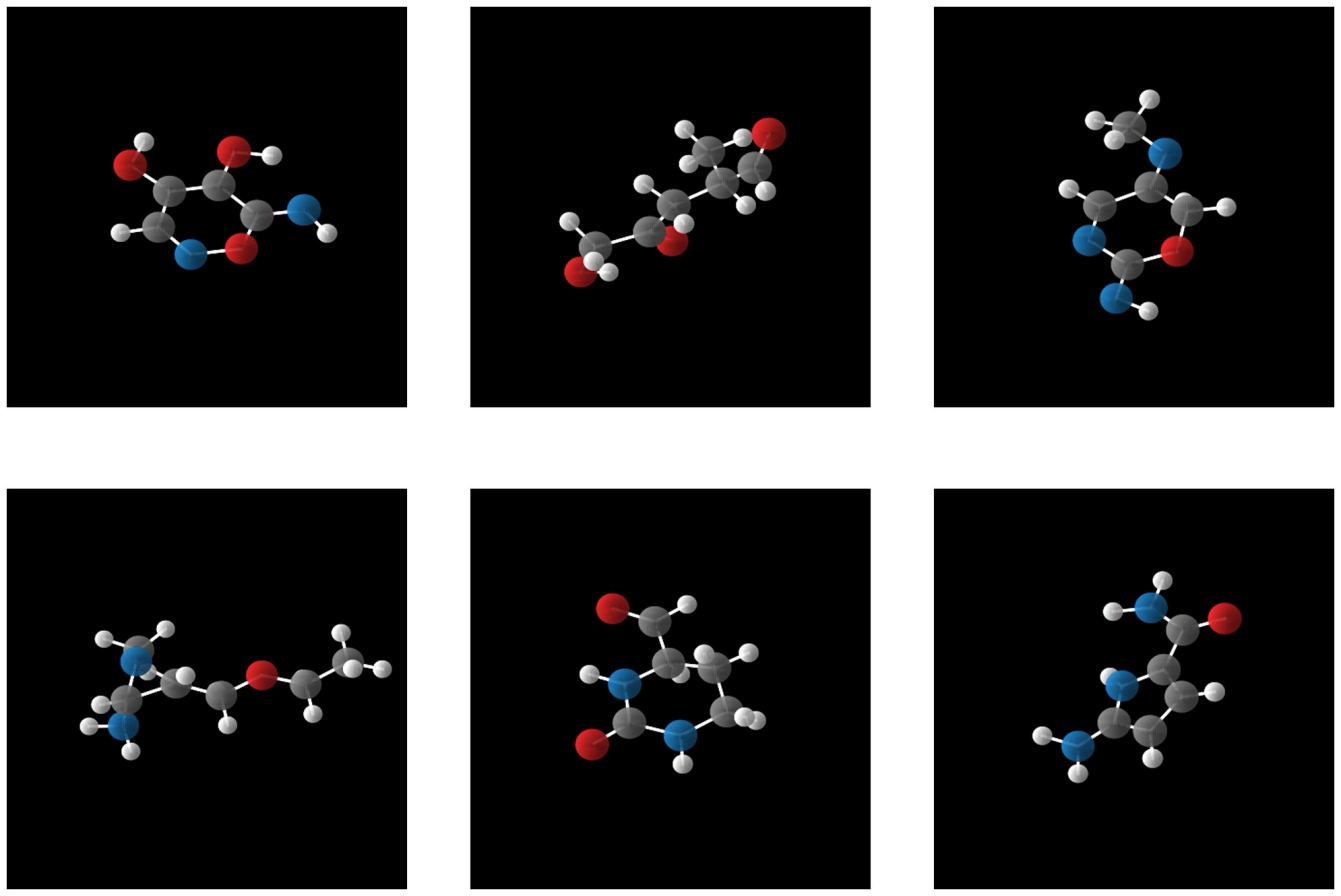
The molecules obtained by randomly sampling from GCLDM under unconditional constraints.

GCLDM is trained without any conditional constraints, allowing the model to learn the distribution of all samples in the training set without focusing on the various attributes of the training samples. After training, molecules are sampled from GCLDM, and the results are shown in Fig. 3.

#### 3.4.2 Conditional generation

**Figure 4.**
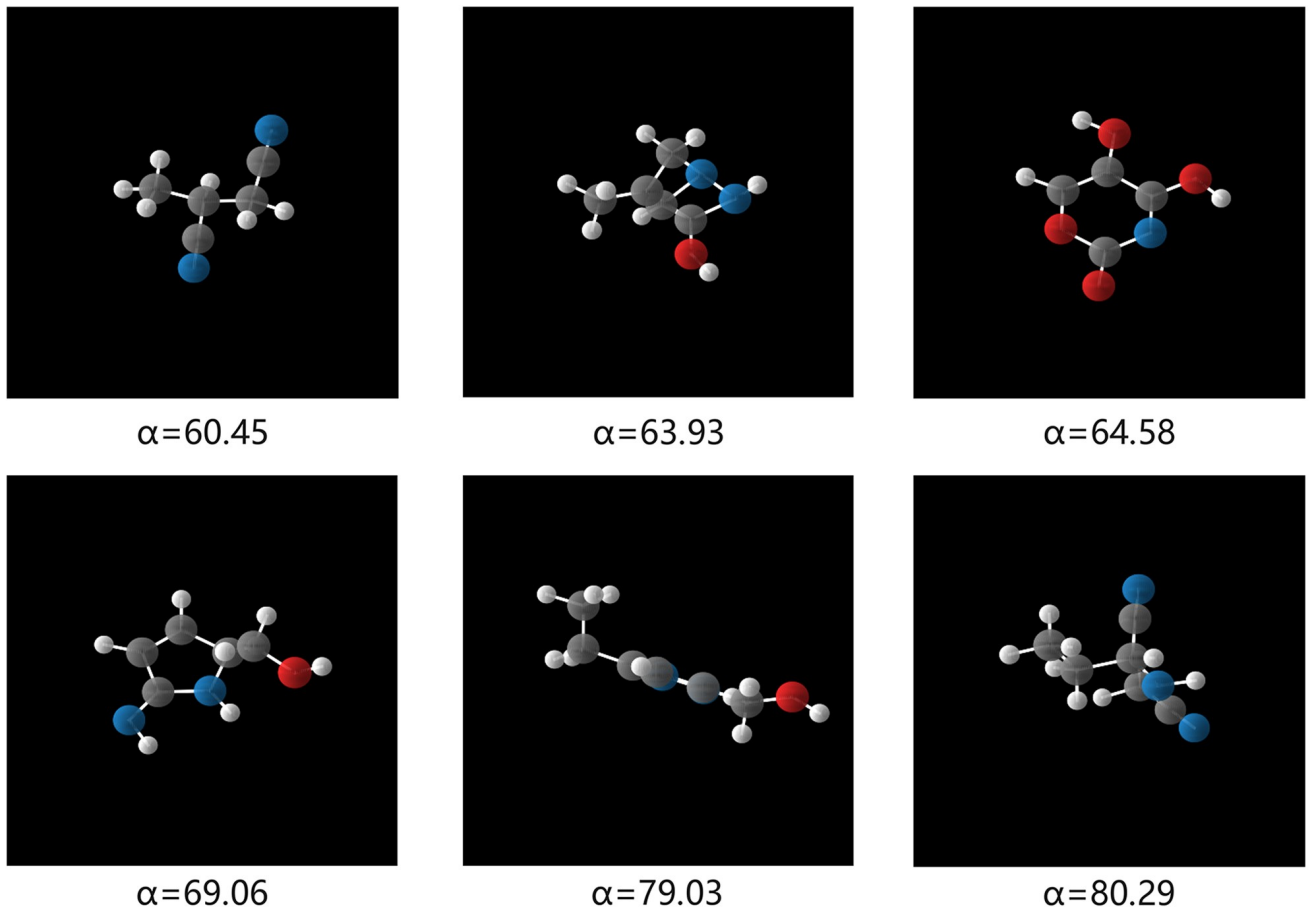
The results of sampling from GCLDM under the given polarizability conditions.

Taking the polarizability α as an example, we train GCLDM with α as the condition. After training, by providing a set of α values as the conditional constraint, the samples generated by GCLDM under this condition are shown in Fig. 4.We visually demonstrate the generated molecules from both unconditional and conditional generation experiments. Since the diffusion model requires multiple samplings of Gaussian noise from a standard normal distribution during the sampling process, and the results are not influenced by human intervention, the results shown reflect randomness and diversity of generated molecules, thereby proving that GCLDM could generate high-quality molecules.

#### 3.4.3 Visualization of denoising process

**Figure 5.**
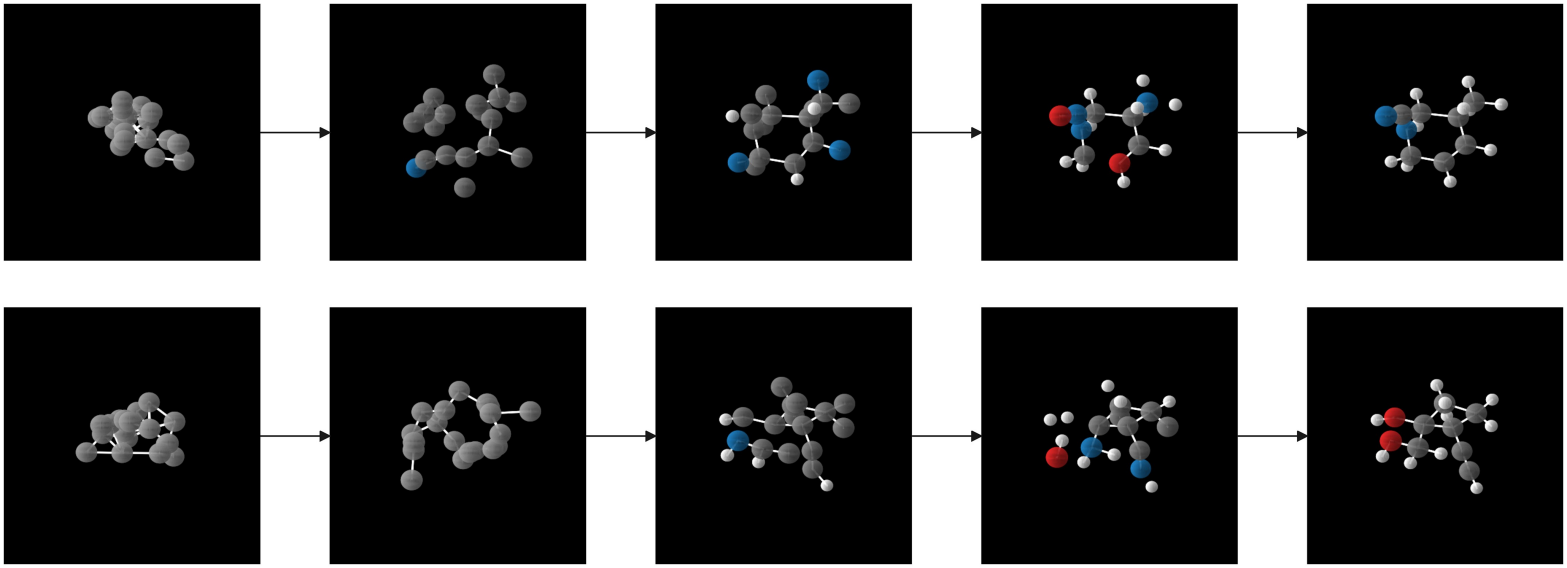
The denoising steps of GCLDM.

The diffusion model progressively denoises through a Markov chain, step by step, to obtain the generated molecules. We also visualized the state of the robust molecules at different time steps during the denoising process to demonstrate how the diffusion model transforms noise into real samples. The results are shown in Fig. 5.

During the sampling process, the model begins by sampling random noise from a standard normal distribution as the starting point for denoising. Therefore, the coordinates of the original noise are always centered around the origin of the 3D coordinate system, which explains why the raw noise in the left side tends to cluster around the center. As the reverse diffusion progresses, the atoms, initially clustered around the origin, gradually diffuse outward. Simultaneously, the features of the atoms become increasingly clearer. Eventually, each atom is restored to its correct position and type. Based on the atom types and bond lengths between atoms, the model predicts the chemical bonds between atom pairs.

## 4 Discussion and Conclusion

The GCLDM, a latent diffusion model for molecule generation, is proposed in this paper. By introducing an autoencoder, the multi-modal features could be mapped to continuous latent representations, thus solving the problem that a unified Gaussian diffusion framework cannot accurately fit the multi-modal feature distributions. On the other hand, we enhance the diffusion model’s denoising capability for 3D geometric structures through a geometry-complete message passing mechanism. Experimental results show that the molecules generated by GCLDM exhibit superior modeling ability in unconditional and conditional molecule generation tasks.

## Data Availability

Our codes and data are all provided at: [https://github.com/charlotte0104/GCLDM-for-3d-molucule-generation] and [https://zenodo.org/records/15773195].
